# Assessment of Differences in Colorectal Cancer Outcomes by Geographic Region for Black Patients in the United States

**DOI:** 10.1007/s40615-025-02455-0

**Published:** 2025-04-28

**Authors:** Mohammed O. Suraju, Darren M. Gordon, Erica Maduakolam, Jordan Grimmett, Alexander Troester, Hassan Aziz, Vincent Reid, Paolo Goffredo, Imran Hassan, Christopher Iverson

**Affiliations:** 1 Department of Surgery, University of Iowa Carver College of Medicine, Iowa City, USA; 2 Department of Internal Medicine, University of Iowa Carver College of Medicine, 200 Hawkins Drive, Iowa City, IA 1516 JCP52242 USA; 3 Division of Colon & Rectal Surgery, University of Minnesota, Minneapolis, MN USA; 4 Department of Surgery, Mercy Hospital, Cedar Rapids, IA USA

**Keywords:** Colon cancer, Rectal cancer, Disparities, Survival

## Abstract

**Introduction:**

Black patients have the worst survival outcomes from colorectal cancer (CRC) in the US. In addition, disparities and differences in mortality outcomes among Black and NHW patients across the four US census regions (Northeast [NE], South, West, Midwest [MW]) remain unexplored. We hypothesized that survival outcomes for Black patients would differ across the US census regions and might correlate with socioeconomic factors.

**Methods:**

Black and Non-Hispanic White (NHW) patients ≥ 45 years of age with a diagnosis of colon or rectal adenocarcinoma between 2010 and 2018 were identified in the National Cancer Database for survival analysis. Survival differences were further validated using the Surveillance, Epidemiology, and End Results (SEER) database to investigate 5-year cause-specific survival (CSS).

**Results:**

For colon adenocarcinoma, the largest difference in median overall survival (OS) between NHW and Black patients was in the MW (67 months Black vs. 74 months NHW, *P* < 0.001). For rectal cancer, the largest difference was in the West (60 months Black vs. 84 months NHW, *P* < 0.001). Black patients receiving care in the MW had the lowest median OS for CRC, while those in the NE had the highest (colon: 67 months MW vs. 100 months NE; rectum: 55 months MW vs. 79 months NE). In multivariable analyses of the Black patient cohort, cancer care in the NE was associated with decreased mortality risk compared to other regions.

**Conclusion:**

Geographic region of care appears to correlate with survival differences for CRC. Exploring these differences may facilitate improved understanding of systemic and structural drivers of health inequities and aid improved resource allocation.

**Supplementary Information:**

The online version contains supplementary material available at 10.1007/s40615-025-02455-0.

## Introduction

Colorectal cancer (CRC) is the second leading cause of cancer deaths in the US, and several studies have highlighted racial disparities in mortality at county and state levels, with Black patients experiencing the worst outcomes [1–4]. For example, in the state of Georgia, a recent study published in *Nature* revealed that Black residents comprised a larger proportion of “CRC hotspot” counties (defined as counties with disease mortality in the fifth quintile) as compared to non-CRC hotspots (41.9% vs. 27.5%) [1]. At the state level, data compiled by the National Institute of Health (NIH) have also highlighted the disparities in CRC outcomes for Black residents [5, 6]. Moreover, the United States Preventive Services Task Force (USPSTF) recommends screening for most average risk individuals to begin at 50 years (Grade A), though starting earlier at age 45 (Grade B) may be considered [7]. In Georgia, the 5-year average CRC mortality rate for Black residents ≥ 50 years old is 55 per 100,000, compared to the rate of 44 per 100,000 among non-Hispanic White (NHW) residents [5]. Notably, Arkansas has the highest 5-year average CRC mortality for Black residents ≥ 50 years old at 74 per 100,000 (compared to 46 per 100,000 for NHW) [5].

An additional level of geographical disparity that has received less attention is the regional level. Regions within the US are defined by the US Census Bureau as Northeast, Midwest, South and West [8, 9]. Of note, these regions are characterized by differing densities of racial/ethnic minorities, and varying degrees of residual structural/systemic underpinnings of slavery and the Jim Crow era that have shaped region-specific socioeconomic disparities (e.g., income, educational attainment etc.) that continue to drive health inequities [10, 11]. Thus, exploring differences and disparities in cancer outcomes at this level has the potential to facilitate improved understanding of systemic and structural bases of health inequities, track progress, and improve resource allocation to address health inequities [12].

Although regional disparities in patient outcomes have been investigated for other diseases, they have yet to be thoroughly explored for oncological diseases such as CRC in the US [13, 14]. We aimed to evaluate differences in survival outcomes for Black patients with CRC across the four US regions and compare disparities to NHW. We hypothesized that survival outcomes for Black patients would differ between regions and potentially correlate with socioeconomic factors.

## Methods

### Data Sources and Study Subjects

The National Cancer Database (NCDB) is a joint program of the Commission on Cancer (CoC) of the American College of Surgeons, and the American Cancer Society and collects approximately 70% of cancer diagnosis in the US. This study was deemed exempt from institutional review board review as patient, hospital, and healthcare provider data were all de-identified. Inclusion criteria were a diagnosis of stage I–IV colon or rectal cancer (excluding the rectosigmoid and appendix) between 2010–2018 (Supplemental Figs. 1 and 2). US census regions as described by the US Census Bureau were utilized [15, 16]. The Northeast included Connecticut, Massachusetts, Maine, New Haven, Rhode Island, Vermont, New Jersey, New York and Pennsylvania. The Midwest comprised of Illinois, Indiana, Michigan, Ohio, Wisconsin, Iowa, Kansas, Minnesota, Missouri, North Dakota, Nebraska and South Dakota. The South included District of Columbia, Delaware, Florida, Georgia, Maryland, North Carolina, Virginia, West Virginia, Alabama, Kentucky, Mississippi, Tennessee, Arkansas, Louisiana, Oklahoma and Texas. The West encompassed Arizona, Colorado, Idaho, New Mexico, Nevada, Utah, Wyoming, Alaska, California, Hawaii, Oregon and Washington state. To validate findings from the NCDB, we assessed cause-specific survival (CSS) in the same period using the Surveillance, Epidemiology, and End Results (SEER) Limited Field Research Data (November 2022 submission), which encompasses approximately 41.9% of the U.S and is curated independently of the NCDB [17]. In contrast to NCDB which is a hospital-based registry, SEER is a population-based registry and collects data from Alaska, California, Connecticut, Massachusetts, Georgia, Hawaii, Idaho, Iowa, Illinois, Kentucky, Louisiana, New Jersey, New Mexico, New York, Washington state, Texas, and Utah.

### Statistical Analyses

Variables used in the analysis were selected a priori based on previous literature, clinical knowledge, and availability. Variables assessed included age, sex, facility type, race, insurance status, income median, neighborhood high school education prevalence, tumor site, tumor size, receipt of chemotherapy, receipt of surgery, surgical margin status, Charlson-Deyo comorbidity Index, Tumor-Node-Metastasis (TNM) stage (a validated cancer staging system), hospital length of stay (LOS), distance from a hospital, number of regional nodes examined, rural residence, surgical approach and re-admission within 30 days. NHW was selected as the reference group to explore disparities in keeping with previous literature given they have the best relative survival, are the most socially advantaged group and are robustly represented in the NCDB [18–23]. As reported by the NCDB, the neighborhood high school education prevalence (NO_HSD_QUAR_00) variable reflects the proportion of adults in the patient’s zip code who did not graduate from high school, categorized in equally proportioned quartiles among all US zip codes (≥ 29%,20–28.9%, 14–19.9% and < 14%). For analytical simplicity, this variable was dichotomized, with the two lower quartiles (i.e., < 14% and 14–19.9%) classified as “above median”, and the higher quartiles as “below median”. Hospital distance was dichotomized at a 30-mile threshold, consistent with prior studies, as this distance often marks a point at which travel may pose logistical challenges to timely and accessible care [24–28]. The 2018 NCDB data dictionary is publicly available (https://www.facs.org/media/12ojeqco/puf_data_dictionary_2018.pdf). Comparison between variables were performed using the chi-square test (categorical variable), student t-test (continuous variables), or mood median test (continuous variables). Unadjusted survival comparison was compared with the Kaplan–Meier method and log-rank test. Adjusted survival analysis was performed using Cox regression model. Covariate estimates are reported as hazard ratios (HRs) along with 95% confidence intervals (95% CIs). All tests were two-sided and assessed for significance at < 5% level. All analyses were conducted using RStudio (R 4.3.2, Vienna, Austria).

## Results

### Black versus Non-Hispanic White Colon Cancer Survival across four US Regions

**Fig. 1:**
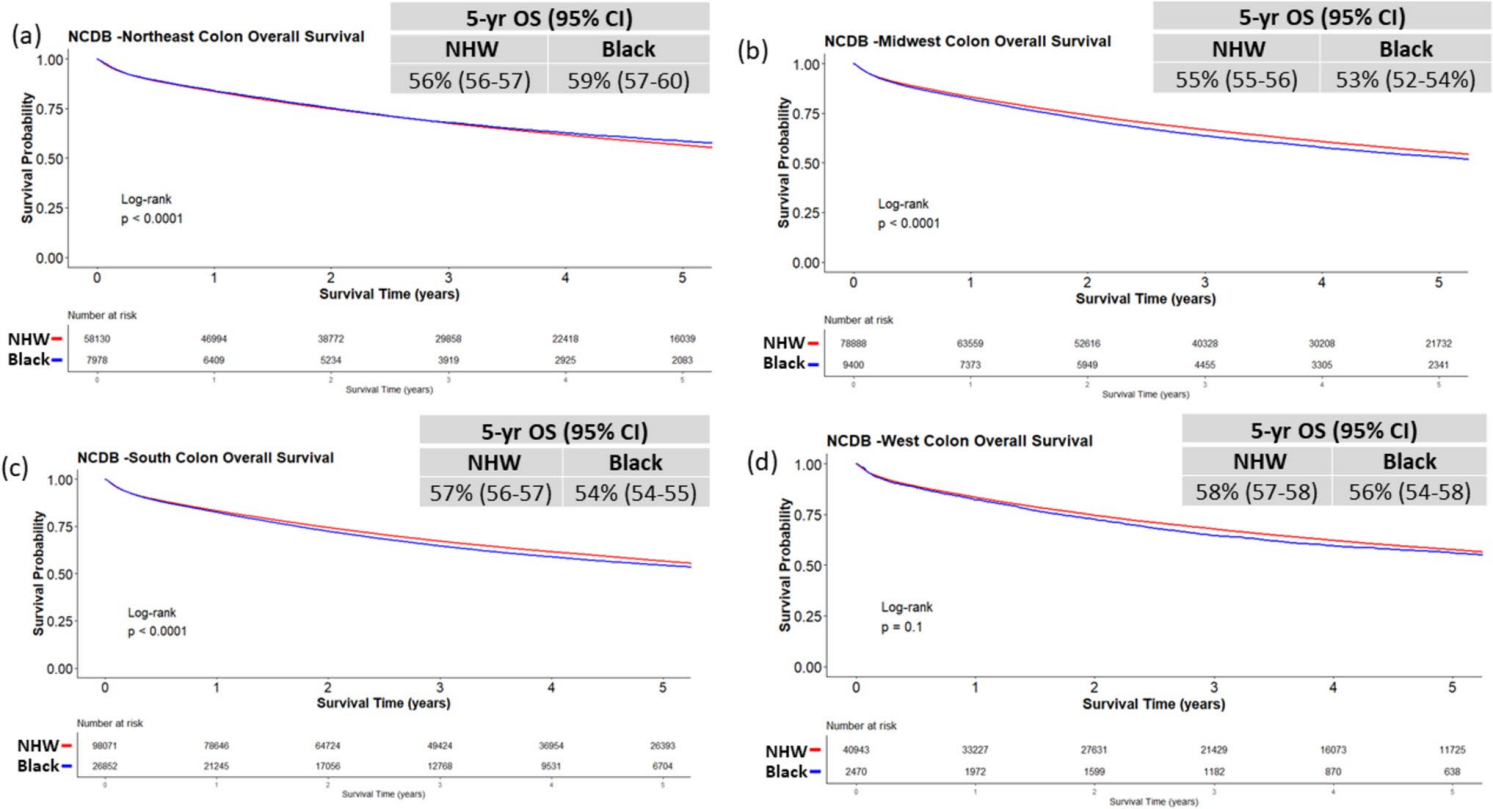
Overall survival for Non-Hispanic White (NHW) and Black patients with colon cancer in each of the four US regions in the NCDB

Of 1,042,794 patients with a diagnosis of colon cancer, 364,611 (311,618 NHW; 52,993 Black) met inclusion criteria for the colon cancer cohort. Among the included patients, there were 88% (65,385) NHW and 12% (8,995) Black that received care in the Northeast; 89% (88,948) NHW and 11% (10,653) Black in the Midwest; 78% (111,063) NHW and 22% (30,527) Black in the South; and 94% (46,222) NHW and 6% (2,818) Black in the West. Across all regions, a greater proportion of Black patients were diagnosed with colon cancer before the age of 70 years, earned below median income, resided in neighborhoods with high school graduation prevalence below the median and had later stage disease at diagnosis (Supplemental Tables 1–4). A greater proportion of Black patients were also treated at academic centers, with the largest proportion being treated in the Northeast (69% Black vs. 44% NHW). Across all regions except the Northeast, Black patients had a lower 5-year overall survival (OS), with the lowest being in the Midwest at 53% (95% CI: 52–54). Five-year OS was less variable for NHW across the four regions (Fig. 1). The greatest disparity in median OS was in the Midwest, where Black and NHW patients had survival of 67 months (95% CI: 64–71) and 74 months (95% CI: 73–75), respectively. Similarly, using the SEER database, colon cancer CSS was found to be lowest in the Midwest with Black patients having a 5-year CSS of 60% compared to 69% for NHW (Supplemental Fig. 3).

### Black Versus NHW Rectal Cancer Survival Across Four US Regions

**Fig. 2:**
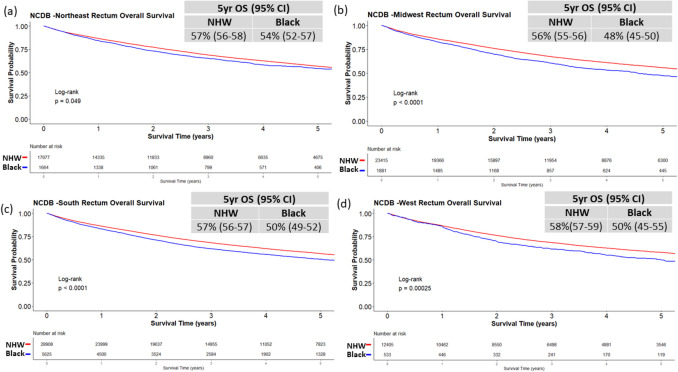
Overall survival for Non-Hispanic White (NHW) and Black patients with rectal cancer in each of the four US regions in the NCDB

Of 340,505 patients with a diagnosis of rectal cancer, 103,422 (92,426 NHW; 10,996 Black) met inclusion criteria for the rectal cancer cohort. The included cohort comprised 91% (19,353) NHW and 9% (1,882) Black in the Northeast; 93% (26,424) NHW and 7% (2,130) Black in the Midwest; 84% (32,692) NHW and 16% (6,381) Black in the South; and 96% (13,957) NHW and 4% (603) Black in the West. Demographic distributions were similar to colon cancer (supplemental Table 5–8). Across all regions, Black patients had a lower 5-year OS, with the lowest being in the Midwest at 48% [95% CI: 45–50]. Again, 5-year OS was less variable for NHW across the four regions (Fig. 2). The greatest disparity in median OS was in the West where Black and NHW had survival of 60 months [95% CI: 50–72] and 84 months [95% CI: 79–87] respectively. The trends in survival were similar to findings in the SEER database, though there were too few Black patients with rectal cancer in the Midwest to detect a significant difference (Supplemental Fig. 4). The lowest rectal cancer CSS was in the South where CSS was 56% for Black patients compared to 66% for NHW patients.

### Survival of Black Patients with Colon Cancer Between Regions

Among Black patients in the colon cancer cohort, those in the Midwest had the lowest median OS, while Black patients in the Northeast had the highest (67 months [95% CI: 64–71] for MW *vs.* 101 months [95% CI: 94–108] for NE). Compared to those in Northeast (Table 1), Black patients in the Midwest had a greater proportion treated at non-academic centers (44% MW *vs.* 31% NE), undergoing surgery with open technique (51% MW *vs.* 44% NE), earning below the median income (55% MW *vs.* 46% NE), and more often residing in rural locations (2.8% MW vs. 0.7% NE). In multivariable analysis, Black patients in the Northeast had decreased risk of mortality compared to Black patients in all other regions (MW *vs.* NE: HR:1.26 [1.19–1.33], *P* < 0.001; S *vs.* NE: HR:1.23 [1.17–1.29], *P* < 0.001, W *vs.* NE: HR: 1.21 [1.11–1.31], *P* < 0.001). Other factors associated with mortality included age, sex, comorbidities, rurality, insurance, tumor characteristics, income, as well as receipt of surgery and chemotherapy (Table 2). For NHW patients (Supplemental Table 9), cancer care in the Northeast was associated with decreased risk of mortality compared to all regions except the West (MW *vs.* NE: HR:1.09[1.07–1.11], *P* < 0.001; S *vs.* NE: HR:1.03[1.01–1.05], *P* < 0.01, W *vs.* NE: HR: 0.99[0.97–1.02], *P* = 0.69).

**Table 1:** Clinicodemographic characteristics of Black Americans diagnosed with colon cancer between 2010–2018 across the four US regions in the NCDB

	Northeast (N = 8995)	Midwest (N = 10,653)	South (N = 30,527)	West (N = 2818)	*P-*value
AGE > 70 years (%)	3553 (39.5)	4018 (37.7)	10,431 (34.2)	1096 (38.9)	< 0.001
SEX, Female (%)	4894 (54.4)	5633 (52.9)	15,891 (52.1)	1424 (50.5)	< 0.001
FACILITY TYPE					< 0.001
Community	628 (7.9)	931 (12.1)	1830 (7.7)	251 (10.8)	
Comprehensive	1848 (23.3)	2464 (32.1)	12,847 (54.2)	1228 (52.8)	
Academic	5471 (68.8)	4288 (55.8)	9011 (38.0)	847 (36.4)	
HOSPITAL DISTANCE > 30 miles (%)	254 (3.0)	333 (3.5)	4077 (15.2)	146 (5.5)	< 0.001
INSURANCE STATUS (%)					< 0.001
None	210 (2.4)	397 (3.8)	2241 (7.5)	68 (2.4)	
Private	2929 (33.2)	3100 (29.7)	9683 (32.2)	1057 (37.7)	
Non-private	5691 (64.5)	6926 (66.4)	18,145 (60.3)	1679 (59.9)	
BELOW MEDIAN INCOME (%)	3757 (45.3)	5119 (54.2)	15,021 (57.5)	837 (33.5)	< 0.001
NEIGHBORHOOD HIGH SCHOOL EDUCATION PREVALENCE > Median(%)	2789 (33.7)	3465 (36.7)	7968 (30.5)	1094 (43.7)	< 0.001
TUMOR PRIMARY SITE, Right (%)	5962 (66.3)	7126 (66.9)	20,257 (66.4)	1924 (68.3)	0.16
TUMOR SIZE > Median (%)	4620 (51.4)	5712 (53.6)	16,272 (53.3)	1580 (56.1)	< 0.001
CHEMOTHERAPY (%)					< 0.001
None	6028 (67.0)	7215 (67.7)	19,751 (64.7)	1926 (68.3)	
Neoadjuvant	129 (1.4)	176 (1.7)	420 (1.4)	41 (1.5)	
Adjuvant	2838 (31.6)	3262 (30.6)	10,356 (33.9)	851 (30.2)	
SURGICAL MARGINS, Positive (%)	403 (5.4)	495 (5.5)	1665 (6.3)	149 (6.2)	< 0.01
CHALSON DEYO SCORE					< 0.001
0	5870 (65.3)	6821 (64.0)	20,337 (66.6)	2004 (71.1)	
1	2045 (22.7)	2330 (21.9)	6768 (22.2)	499 (17.7)	
≥ 2	1080 (12.0)	1502 (14.1)	3422 (11.2)	315 (11.2)	
TNM STAGE (%)					< 0.001
1	2065 (23.0)	2523 (23.7)	6583 (21.6)	632 (22.4)	
2	2079 (23.1)	2504 (23.5)	7522 (24.6)	689 (24.4)	
3	2594 (28.8)	2982 (28.0)	8997 (29.5)	844 (30.0)	
4	2257 (25.1)	2644 (24.8)	7425 (24.3)	653 (23.2)	
SURGICAL APPROACH (%)					< 0.001
Minimally Invasive (MIS)	3422 (48.6)	3574 (42.0)	10,758 (44.0)	1172 (51.2)	
MIS to open	555 (7.9)	641 (7.5)	1616 (6.6)	139 (6.1)	
Open	3061 (43.5)	4293 (50.5)	12,094 (49.4)	977 (42.7)	
REGIONAL NODES EXAMINED ≥ 12 (%)	6511 (73.1)	7738 (73.4)	22,581 (74.7)	2093 (74.8)	0.002
READMISSION WITHIN 30 DAYS, Yes (%)	581 (6.6)	573 (5.4)	1487 (4.9)	66 (2.3)	< 0.001
RURAL RESIDENTS (%)	61 (0.7)	298 (2.8)	4608 (15.3)	16 (0.6)	< 0.001
NO EXCISION (%)	1439 (16.2)	1573 (14.9)	3856 (12.8)	388 (13.9)	< 0.001

**Table 2:** Multivariable model of factors associated with colon cancer mortality among Black patients across the four US regions in the NCDB

	Variables	Hazard Ratio (95% CI)	*P*-value
AGE	< 70 years	*Ref*	
	≥ 70 years	1.61 (1.55–1.68)	< 0.001
SEX	Male	*Ref*	
	Female	0.88 (0.85–0.91)	< 0.001
METRO	Urban	*Ref*	
	Rural	1.08 (1.01–1.15)	0.03
FACILITY TYPE	Community	*Ref*	
	Comprehensive	1.07 (1.01–1.14)	0.03
	Academic	0.99 (0.94–1.07)	0.96
CHALSON DEYO SCORE	0	*Ref*	
	1	1.10 (1.06–1.15)	< 0.001
	≥ 2	1.40 (1.33–1.47)	< 0.001
INSURANCE	None	*Ref*	
	Private	0.84 (0.78–0.91)	< 0.001
	Non-private	1.11 (1.03–1.20)	< 0.01
HOSPITAL DISTANCE	< 30 miles	*Ref*	
	≥ 30 miles	1.02 (0.95–1.09)	0.58
INCOME	≥ Median	*Ref*	
	< Median	1.04 (1.00–1.08)	0.03
TUMOR SITE	Left	*Ref*	
	Right	1.11 (1.07–1.15)	< 0.001
TUMOR SIZE	< Median	*Ref*	
	≥ Median	1.06 (1.02–1.10)	0.002
REGIONAL NODES EXAMINED	< 12	*Ref*	
	≥ 12	0.68 (0.64–0.71)	< 0.001
TNM STAGE	1	*Ref*	
	2	1.55 (1.45–1.65)	< 0.001
	3	3.21 (3.00–3.43)	< 0.001
	4	10.0 (9.36–10.8)	< 0.001
TUMOR EXCISION	Yes	*Ref*	
	No	1.13 (1.05–1.22)	0.001
CHEMOTHERAPY	None	*Ref*	
	Neoadjuvant	0.56 (0.49–0.65)	< 0.001
	Adjuvant	0.55 (0.52–0.58)	< 0.001
READMISSION	No	*Ref*	
	Yes	1.43 (1.33–1.54)	< 0.001
GEOGRAPHIC REGION	Northeast	*Ref*	
	Midwest	1.26 (1.19–1.33)	< 0.001
	South	1.23 (1.17–1.29)	< 0.001
	West	1.21 (1.11–1.31)	< 0.001

### Survival of Black Patients with Rectal Cancer Between Regions

Similar to the colon cancer cohort, Black patients in the rectal cancer cohort in the Midwest had the lowest median OS, while those in the Northeast had the highest (55 months [95% CI: 51–60] MW *vs.* 79 months [95% CI: 66–92] NE). In addition, Black patients with rectal cancer in the Midwest (Table 3) had a greater proportion treated at non-academic hospitals (63% MW *vs.* 70% NE), undergoing surgery with open technique (50% MW *vs.* 46% NE), earning below the median income (57% MW *vs.* 48% NE), and more often residing in rural locations (0.8% MW *vs.* 3.1% NE). In multivariable analysis (Table 4), Black patients receiving care in the NE again had decreased risk of mortality compared to Black patients in all other regions (MW *vs.* NE: HR:1.23 [1.09–1.39], *P* = 0.001; S *vs.* NE: HR:1.18 [1.06–1.31], *P* < 0.01, W *vs.* NE: HR: 1.28 [1.08–1.52], *P* < 0.01). Among NHW patients (Supplemental Table 10), rectal cancer care in the Northeast was also associated with decreased risk of mortality compared to all other regions (MW *vs.* NE: HR:1.15 [1.11–1.20], *P* < 0.001; S *vs.* NE: HR:1.11 [1.07–1.16], *P* < 0.001, W *vs.* NE: HR: 1.07 [1.02–1.12], *P* < 0.01).

**Table 3:** Clinicodemographic characteristics of Black Americans diagnosed with rectal cancer between 2010–2018 across the four US regions in the NCDB

	Northeast (N = 1882)	Midwest (N = 2130)	South (N = 6381)	West (N = 603)	*P-*value
AGE > 70 years (%)	670 (35.6)	698 (32.8)	1862 (29.2)	169 (28.0)	< 0.001
SEX, Female (%)	757 (40.2)	886 (41.6)	2562 (40.2)	249 (41.3)	0.66
FACILITY TYPE					< 0.001
Community	96 (5.8)	127 (8.0)	328 (6.5)	60 (12.4)	
Comprehensive	409 (24.7)	456 (28.7)	2442 (48.1)	197 (40.8)	
Academic	1154 (69.6)	1007 (63.3)	2309 (45.5)	226 (46.8)	
HOSPITAL DISTANCE > 30 miles (%)	64 (3.7)	99 (5.1)	1126 (20.0)	44 (7.8)	< 0.001
INSURANCE STATUS (%)					< 0.001
None	63 (3.4)	86 (4.1)	593 (9.5)	22 (3.7)	
Private	608 (33.0)	656 (31.6)	1995 (31.9)	227 (37.8)	
Non-private	1170 (63.6)	1336 (64.3)	3670 (58.6)	352 (58.6)	
BELOW MEDIAN INCOME (%)	826 (47.9)	1081 (57.0)	3210 (59.0)	183 (34.5)	< 0.001
NEIGHBORHOOD HIGH SCHOOL EDUCATION PREVALENCE > Median(%)	527 (30.6)	666 (35.2)	1538 (28.2)	216 (40.8)	< 0.001
TUMOR SIZE > median (%)	1120 (59.5)	1263 (59.3)	3770 (59.1)	351 (58.2)	0.95
CHEMOTHERAPY (%)					0.29
None	1160 (61.6)	1290 (60.6)	3789 (59.4)	379 (62.9)	
Neoadjuvant	495 (26.3)	598 (28.1)	1786 (28.0)	159 (26.4)	
Adjuvant	227 (12.1)	242 (11.4)	806 (12.6)	65 (10.8)	
RADIATION (%)					0.02
None	1253 (66.6)	1369 (64.3)	4056 (63.6)	397 (65.8)	
Neoadjuvant	493 (26.2)	616 (28.9)	1761 (27.6)	158 (26.2)	
Adjuvant	136 (7.2)	145 (6.8)	564 (8.8)	48 (8.0)	
SURGICAL MARGINS, Positive (%)	110 (10.5)	113 (8.7)	395 (10.1)	35 (9.9)	0.43
CHALSON DEYO SCORE					0.02
0	1343 (71.4)	1516 (71.2)	4610 (72.2)	473 (78.4)	
1	358 (19.0)	400 (18.8)	1205 (18.9)	89 (14.8)	
≥ 2	181 (9.6)	214 (10.0)	566 (8.9)	41 (6.8)	
TNM STAGE (%)					0.24
1	481 (25.6)	573 (26.9)	1710 (26.8)	177 (29.4)	
2	419 (22.3)	471 (22.1)	1452 (22.8)	111 (18.4)	
3	553 (29.4)	575 (27.0)	1719 (26.9)	167 (27.7)	
4	429 (22.8)	511 (24.0)	1500 (23.5)	148 (24.5)	
SURGICAL APPROACH (%)					< 0.001
MIS	463 (47.3)	535 (43.9)	1480 (42.2)	182 (54.8)	
MIS to open	63 (6.4)	77 (6.3)	189 (5.4)	26 (7.8)	
Open	452 (46.2)	607 (49.8)	1840 (52.4)	124 (37.3)	
REGIONAL NODES EXAMINED ≥ 12 (%)	640 (34.8)	822 (39.7)	2366 (38.0)	227 (38.2)	0.02
READMISSION WITHIN 30 DAYS, Yes (%)	107 (5.8)	101 (4.8)	279 (4.4)	17 (2.8)	0.01
RURAL RESIDENTS (%)	14 (0.8)	64 (3.1)	1071 (17.2)	6 (1.0)	< 0.001
NO EXCISION (%)	776 (42.4)	772 (37.8)	2331 (37.8)	225 (38.2)	< 0.01

**Table 4:** Multivariable model of factors associated with rectal cancer mortality among Black patients across the four US regions in the NCDB

	Variables	Hazard Ratio (95% CI)	*P*-value
AGE	< 70	*Ref*	
	≥ 70	1.68 (1.55–1.82)	< 0.001
SEX	Male	*Ref*	
	Female	0.87 (0.81–0.94)	< 0.001
METRO	Urban	*Ref*	
	Rural	1.01 (0.88–1.16)	0.88
FACILITY TYPE	Community	*Ref*	
	Comprehensive	1.13 (0.98–1.31)	0.09
	Academic	0.99 (0.86–1.15)	0.91
CHALSON DEYO SCORE	0	*Ref*	
	1	1.14 (1.04–1.25)	< 0.01
	≥ 2	1.61 (1.43–1.82)	< 0.001
INSURANCE	None	*Ref*	
	Private	0.91 (0.78–1.05)	0.19
	Non-Private	1.16 (1.01–1.33)	0.04
HOSPITAL DISTANCE	< 30 miles	*Ref*	
	≥ 30 miles	0.99 (0.87–1.13)	0.91
INCOME	≥ Median	*Ref*	
	< Median	1.10 (1.02–1.18)	0.02
TUMOR SIZE	< Median	*Ref*	
	≥ Median	1.02 (0.95–1.11)	0.57
NUMBER OF NODES EXAMINED	< 12	*Ref*	
	≥ 12	0.85 (0.76–0.96)	< 0.01
TNM STAGE	1	*Ref*	
	2	1.70 (1.48–1.95)	< 0.001
	3	2.06 (1.80–2.36)	< 0.001
	4	4.96 (4.32–5.69)	< 0.001
TUMOR EXCISION	Yes	*Ref*	
	No	1.60 (1.39–1.85)	< 0.001
CHEMOTHERAPY	None	*Ref*	
	Neoadjuvant	0.79 (0.64–0.98)	0.03
	Adjuvant	0.78 (0.65–0.94)	< 0.01
RADIATION	None	*Ref*	
	Neoadjuvant	0.90 (0.73–1.11)	0.32
	Adjuvant	0.89 (0.73–1.08)	0.23
READMISSION	No		
	Yes	1.44 (1.19–1.74)	< 0.001
GEOGRAPHIC REGION	Northeast	*Ref*	
	Midwest	1.23 (1.09–1.39)	0.001
	South	1.18 (1.06–1.31)	0.002
	West	1.28 (1.08–1.52)	0.005

## Discussion

In this study, we explored regional disparities in survival outcomes for Black patients with CRC across the four US census regions. We found that disparities in median and 5-year survival for each region were larger for rectal cancer than for colon cancer, possibly reflecting the differences in the complexity of management for both diseases. Black CRC patients had worse OS compared to NHW, the exceptions were for colon cancer where NHW had worse OS in the NE and similar OS in the West. Additionally, while OS and CSS appeared to be similar across all four regions for NHW patients, for Black patients, they were more varied. Among Black patients, those receiving CRC cancer in the Northeast had the best 5-year OS for CRC, while those in the Midwest had the worst.

Compared to Black patients in the Northeast, those in the Midwest had a lower proportion treated at academic centers, earning above the median income, and residing in non-rural areas. These factors may partly explain the poorer outcomes observed in the Midwest. Of note, below median income was consistently independently associated with increased mortality for both colon and rectal cancer, and its prevalence was appreciably higher among patients in the Midwest. Notwithstanding, the impact of other influential factors not captured in our dataset but known to disproportionately impact minority communities, such as, smoking prevalence (14% MW vs. 10% NE), obesity prevalence (36% MW vs. 28.6% NE), access to healthy food options, screening uptake, as well as local and state policies should not be overlooked [10, 29–31]. Moreover, the Midwest faces significant challenges in healthcare workforce diversity which has been linked to disparities in healthcare accessibility and outcomes [32, 33]. It is worth noting that although there may be differences in density of hospital expertise across regions, it is an unlikely explanation for the disparities observed in our study, as OS and CSS were relatively similar among NHW patients across all four regions. Nevertheless, our finding that Black patients in the Northeast exhibit better outcomes for CRC warrants further investigation as it suggests the presence of modifiable regional factors.

Notably, the study by Naishadham et al. (2011) offers valuable context that complements and supports the findings of our study. Briefly, they evaluated the trends in age-standardized mortality rates from CRC for each state between 1990–2007 [6]. They noted that the largest reductions in CRC mortality rates occurred in Northeastern states, while the Southern states had the smallest reductions. Additionally, they found a strong correlation between state screening uptake and decreases in CRC mortality rates (r = − 0.65, *P* < 0.0001). In a subsequent study, the same group modeled predicted outcomes for Louisiana (a southern state) if it achieved similar levels of smoking/obesity risk factors and CRC screening uptake as New Jersey (a Northeastern state) [34]. They estimated that attaining similar levels of smoking/obesity risk factors to New Jersey would reduce CRC mortality by 1.6% for Black residents and 2.5% for White residents in Louisiana. On the other hand, achieving a similar screening uptake to New Jersey would reduce CRC mortality rates by 11.7% for Black residents and 14.7% for White Louisiana residents. The authors emphasized that the excess mortality that persisted for Black residents suggested that other factors remained that contribute to survival differences.

One of the most notable examples of a coordinated public health initiative to address disparities in colorectal cancer screening comes from New York, a Northeastern state [35]. Through its New York Citywide Colon Cancer Control Coalition, overall colonoscopy screening rates increased from 42% in 2003 to 70% in 2014. Among Black residents specifically, screening rates increased from approximately 35% to 70%. This success was driven by a multifaceted approach that included public and provider education campaigns, implementation of patient navigator programs, streamlined endoscopic referral systems, targeted outreach at safety-net hospitals, and free screening services for uninsured individuals. These efforts were funded by the New York City Department of Health and Mental Hygiene (NYC DOHMH), the state government, the Center for Disease Control and Prevention (CDC), and philanthropic contributions. Building on the success of programs like this, the CDC launched the Colorectal Cancer Control Program (CRCCP), providing grant support from 2020–2025 to 20 state health departments to implement similar screening initiatives. Among Midwestern recipients were Iowa, Kansas, Minnesota and Nebraska, and South Dakota [36]. Additionally, eight universities received grants, including the University of Chicago (Illinois) and University of Missouri (Missouri).

Our study should be interpreted within the context of several limitations. Firstly, given its retrospective nature, there is an inherent risk of selection bias. Additionally, cause-specific mortality is not reported in the NCDB. Moreover, NCDB is a hospital-based registry and only patients treated at CoC sites are captured by the NCDB, potentially underestimating disparities as patients treated at non-CoC sites may not be included. To address these limitations, we also utilized the SEER database to validate our findings. While SEER has its own constraints, such as more limited scope, it is population-based, includes non-CoC centers and provides data on cause-specific mortality rates.

In summary, our findings indicate the varying degrees of regional disparities in CRC mortality in the US. These differences highlight that, beyond disease biology, social determinants of health and possibly other unrecognized factors exert strong influences on survival outcomes. Particularly notable is the observation that Black patients with CRC in the Northeast exhibit better survival compared to other regions. Future studies are needed to explore the drivers of this difference and may provide valuable insights for studying national disparities trends and inform resource allocation.

## Supplementary Information

Below is the link to the electronic supplementary material.

Supplementary file1 (DOCX 822 KB)

## Data Availability

The deidentified data utilized for this study are publicly available from the National Cancer and the Surveillance, Epidemiology, and End Results database.

## Code Availability

Code utilized for analysis is available from the corresponding author upon reasonable request.
