# Supplementary material for: Assessment of Differences in Colorectal Cancer Outcomes by Geographic Region for Black Patients in the United States
Source: J Racial Ethn Health Disparities. 2025 Apr 28;13(4):2782–92. doi: 10.1007/s40615-025-02455-0 (PMC13346272; doi:10.1007/s40615-025-02455-0)
Supplement: Supplementary file 1 — Supplementary file1 (DOCX 822 KB) [file 40615_2025_2455_MOESM1_ESM.docx]

**Supplemental Tables and Figures**

**Supplemental Figure 1:** CONSORT diagram of patient selection criteria among colon cancer patients.

**
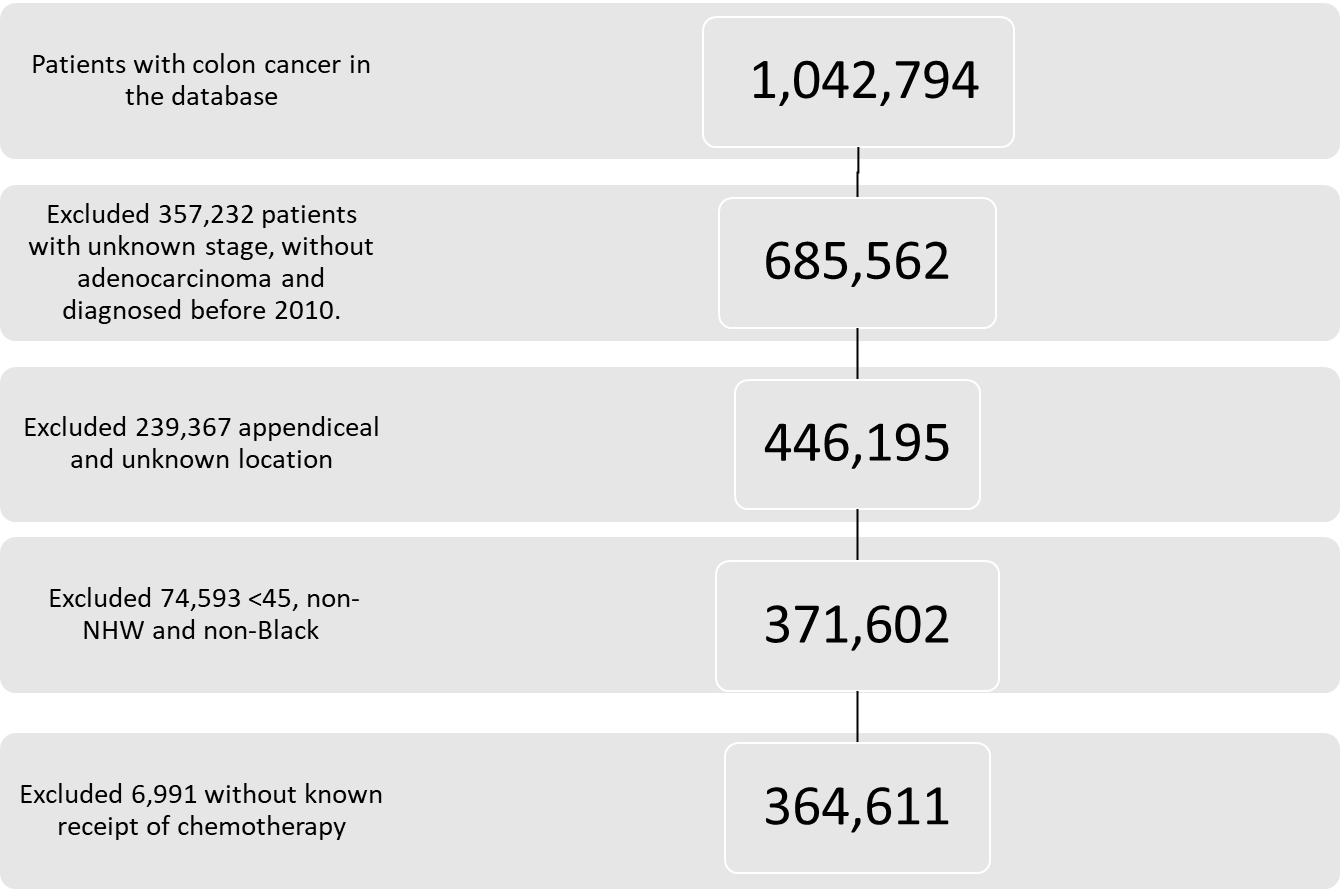
**

**Supplemental Figure 2:** CONSORT diagram of patient selection criteria among rectal cancer patients.


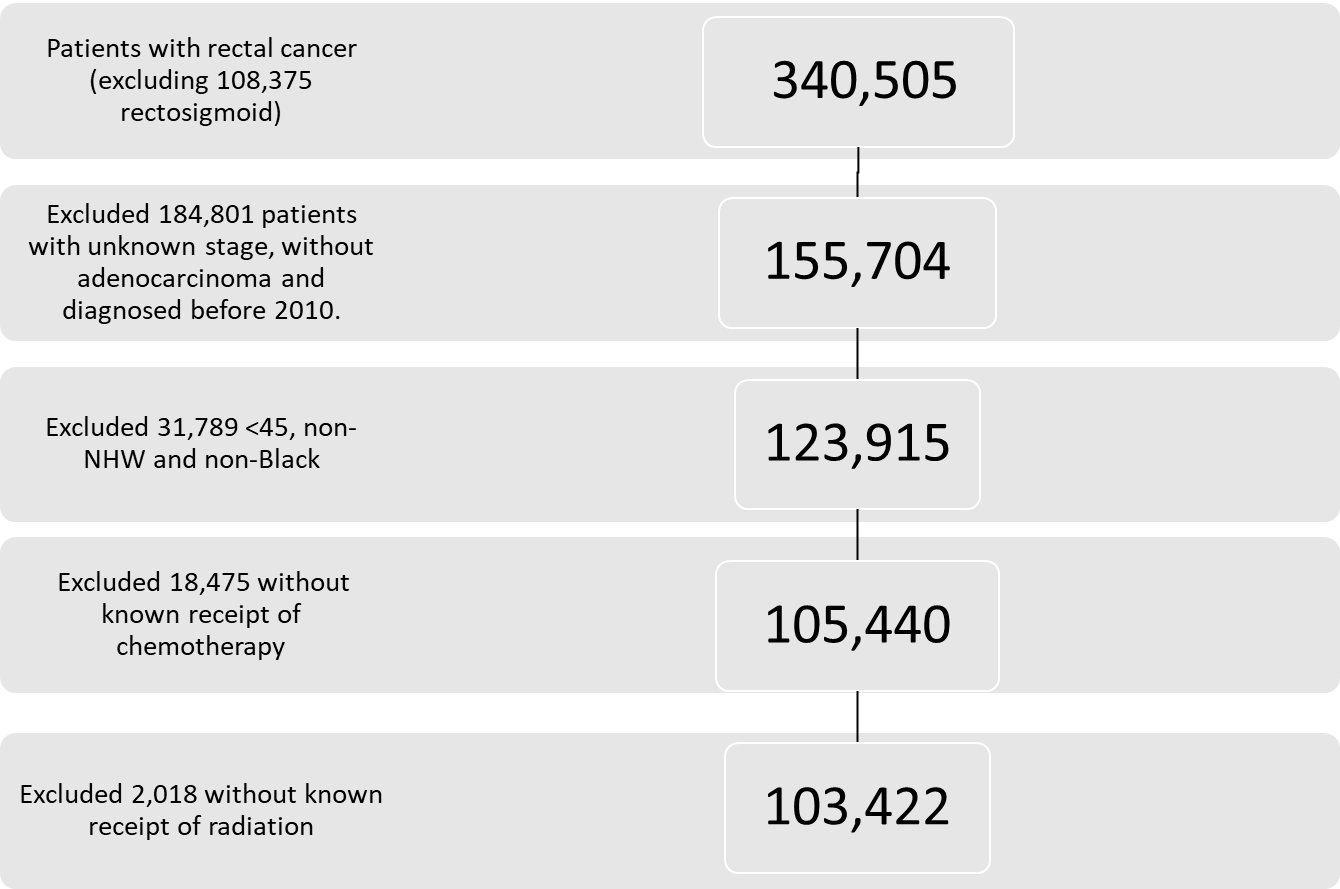


**Supplemental Figure 3:** Cancer-specific survival for Non-Hispanic White (NHW) and Black patients with colon cancer in each of the four US regions using the SEER database.

**
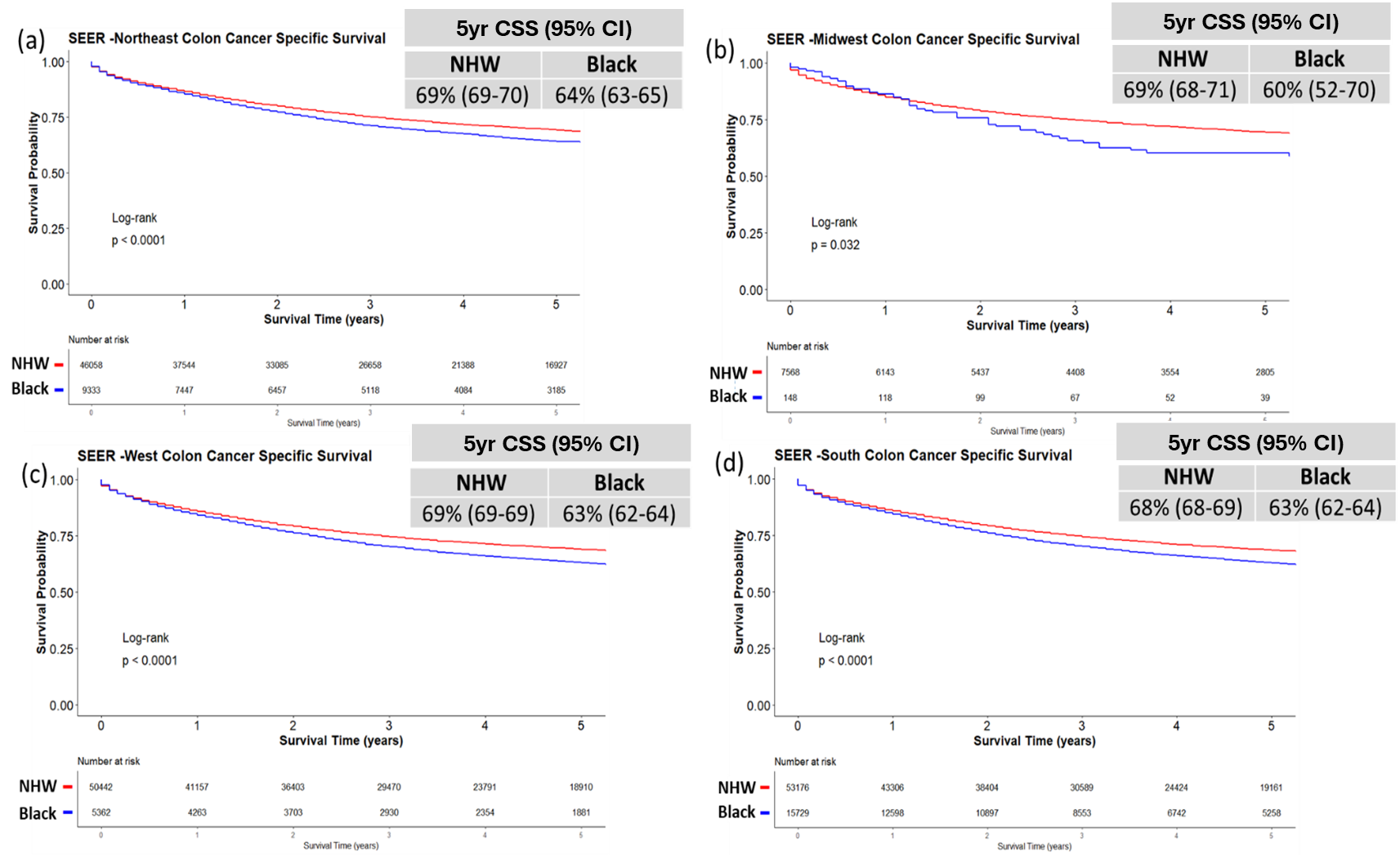
**

**Supplemental Figure 4:** Cancer-specific survival for NHW and Black patients with rectal cancer in each of the four US regions using the SEER database.

**
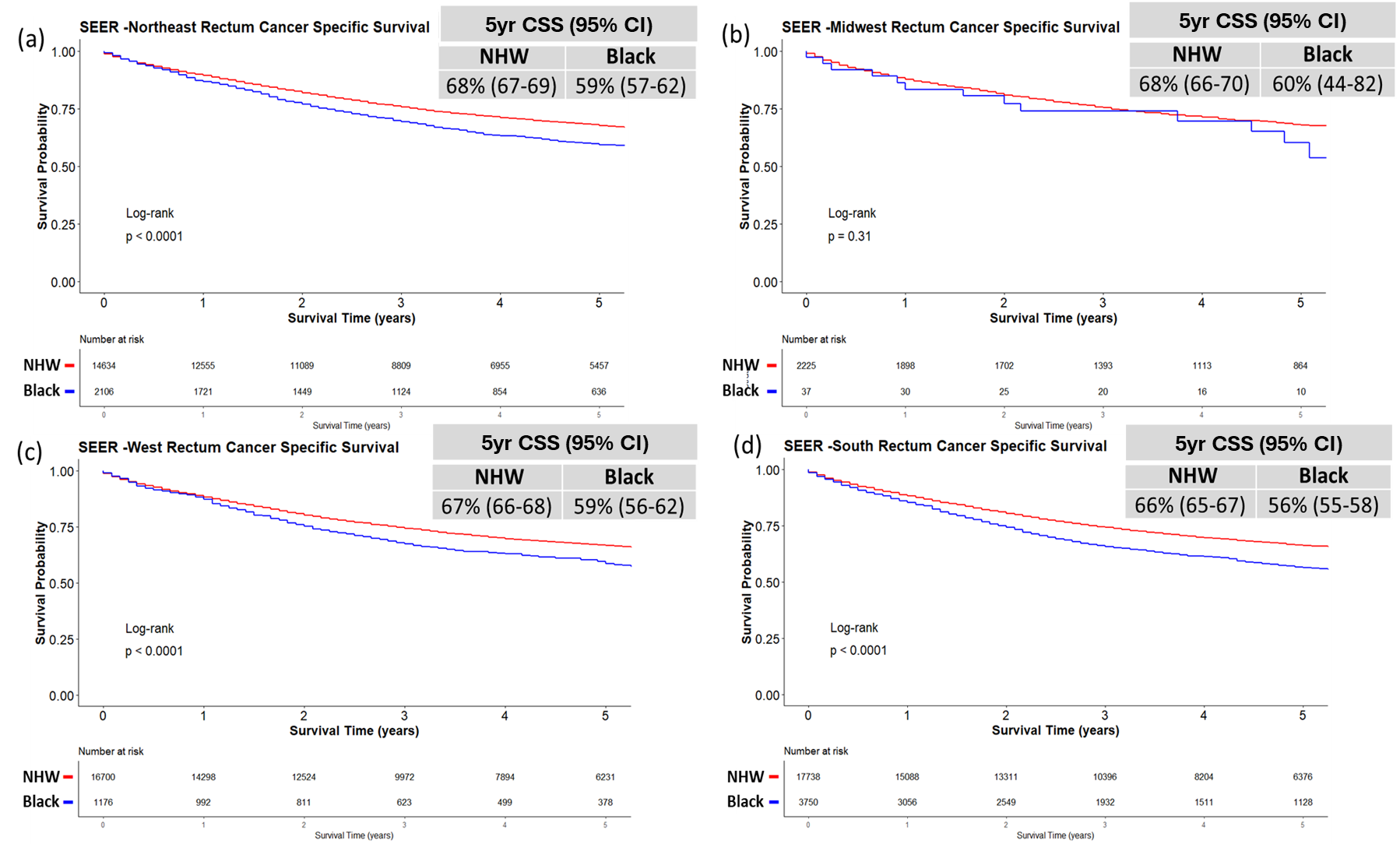
**

**Supplemental Table 1:** Clinicodemographic characteristics of Non-Hispanic White (NHW) and Black Americans diagnosed with colon cancer between 2010–2018 in the Northeast in the NCDB.

|  | **NHW**  **(N=65385)** | **Black**  **(N=8995)** | ***P*-value** |
| --- | --- | --- | --- |
| **AGE ≥70 (%)** | 37059 (57) | 3553 (39.5) | <0.001 |
| **SEX, Female (%)** | 33277 (51) | 4894 (54.4) | <0.001 |
| **FACILITY TYPE** |  |  | <0.001 |
| Community | 5546 (10) | 628 (7.9) |  |
| Comprehensive | 25664 (46) | 1848 (23.3) |  |
| Academic | 24999 (45) | 5471 (68.8) |  |
| **HOSPITAL DISTANCE >30miles (%)** | 6069 (10) | 254 (3.0) | <0.001 |
| **INSURANCE STATUS (%)** |  |  | <0.001 |
| None | 658 (1) | 210 (2) |  |
| Private | 19594 (30) | 2929 (33) |  |
| Non-private | 44211 (69) | 5691 (65) |  |
| **BELOW MEDIAN INCOME (%)** | 10959 (19) | 3757 (45) | <0.001 |
| **NEIGHBORHOOD HIGH SCHOOL EDUCATION PREVALENCE >Median(%)** | 43560 (74) | 2789 (34) | <0.001 |
| **TUMOR PRIMARY SITE, Right (%)** | 42777 (65) | 5962 (66) | 0.111 |
| **TUMOR SIZE >median (%)** | 32443 (50) | 4620 (51) | <0.01 |
| **CHEMOTHERAPY (%)** |  |  | <0.001 |
| None | 45908 (70) | 6028 (67) |  |
| Neoadjuvant | 833 (1) | 129 (1) |  |
| Adjuvant | 18644 (29) | 2838 (32) |  |
| **SURGICAL MARGINS, Positive (%)** | 3111 (5) | 403 (5) | 0.91 |
| **CHALSON DEYO SCORE** |  |  | 0.001 |
| 0 | 43801 (67) | 5870 (65) |  |
| 1 | 13782 (21) | 2045 (23) |  |
| ≥2 | 7802 (12) | 1080 (12) |  |
| **TNM STAGE (%)** |  |  | <0.001 |
| 1 | 16788 (26) | 2065 (23) |  |
| 2 | 18709 (29) | 2079 (23) |  |
| 3 | 17985 (28) | 2594 (29) |  |
| 4 | 11903 (18) | 2257 (25) |  |
| **SURGICAL APPROACH (%)** |  |  | <0.001 |
| MIS | 28306 (53) | 3422 (49) |  |
| MIS to open | 3679 (7) | 555 (8) |  |
| Open | 21924 (41) | 3061 (44) |  |
| **REGIONAL NODES EXAMINED ≥12 (%)** | 51128 (78) | 6511 (72) | <0.001 |
| **READMISSION WITHIN 30-DAYS, Yes (%)** | 3656 (6) | 581(7) | 0.03 |
| **RURAL RESIDENTS (%)** | 5253 (8) | 61 (1) | <0.001 |
| **NO EXCISION (%)** | 6836 (10) | 1439 (16) | <0.001 |

**Supplemental Table 2:** Clinicodemographic characteristics of Non-Hispanic White (NHW) and Black Americans diagnosed with colon cancer between 2010–2018 in the Midwest in the NCDB.

|  | **NHW**  **(N=88948)** | **Black**  **(N=10653)** | ***P*-value** |
| --- | --- | --- | --- |
| **AGE ≥70 (%)** | 48837 (55) | 4018 (38) | <0.001 |
| **SEX, Female (%)** | 44649 (50) | 5633 (53) | <0.001 |
| **FACILITY TYPE** |  |  | <0.001 |
| Community | 11320 (17) | 931 (12) |  |
| Comprehensive | 33086 (51) | 2464 (32) |  |
| Academic | 21003 (32) | 4288 (56) |  |
| **HOSPITAL DISTANCE>30miles (%)** | 15689 (20) | 333 (4) | <0.001 |
| **INSURANCE STATUS (%)** |  |  | <0.001 |
| None | 1502 (2) | 397 (4) |  |
| Private | 25182 (29) | 3100 (30) |  |
| Non-private | 61250 (70) | 6926 (66) |  |
| **BELOW MEDIAN INCOME (%)** | 18384 (23) | 5119 (54) | <0.001 |
| **NEIGHBORHOOD HIGH SCHOOL EDUCATION PREVALENCE >Median(%)** | 58133 (74) | 3465 (37) | <0.001 |
| **TUMOR PRIMARY SITE, Right (%)** | 59272 (67) | 7126 (67) | 0.61 |
| **TUMOR SIZE >median (%)** | 44756 (50) | 5712 (54) | <0.001 |
| **CHEMOTHERAPY (%)** | |  | <0.001 |
| None | 61499 (69) | 7215 (68) |  |
| Neoadjuvant | 1100 (1) | 176 (2) |  |
| Adjuvant | 26349 (30) | 3262 (31) |  |
| **SURGICAL MARGINS, Positive (%)** | 4636 (6) | 495 (6) | 0.39 |
| **CHALSON DEYO SCORE** |  |  | 0.36 |
| 0 | 56790 (64) | 6821 (64.0) |  |
| 1 | 19921 (22) | 2330 (22) |  |
| ≥2 | 12237 (14) | 1502 (14) |  |
| **TNM STAGE (%)** | |  | <0.001 |
| 1 | 22455 (25) | 2523 (24) |  |
| 2 | 25602 (29) | 2504 (24) |  |
| 3 | 25196 (28) | 2982 (28) |  |
| 4 | 15695 (18) | 2644 (25) |  |
| **SURGICAL APPROACH (%)** | |  | <0.001 |
| MIS | 35720 (47) | 3574 (42) |  |
| MIS to open | 5142 (7) | 641 (8) |  |
| Open | 34717 (46) | 4293 (51) |  |
| **REGIONAL NODES EXAMINED ≥12 (%)** | 71552 (81) | 7738 (73) | <0.001 |
| **READMISSION WITHIN 30-DAYS, Yes (%)** | 4469 (5) | 573 (5) | 0.12 |
| **RURAL RESIDENTS (%)** | 21848 (25) | 298 (3) | <0.001 |
| **NO EXCISION (%)** | 7733 (9) | 1573 (15) | <0.001 |

**Supplemental Table 3:** Clinicodemographic characteristics of Non-Hispanic White (NHW) and Black Americans diagnosed with colon cancer between 2010–2018 in the South in the NCDB.

|  | **NHW**  **(N=111063)** | **Black**  **(N=30527)** | ***P*-value** |
| --- | --- | --- | --- |
| **AGE ≥70 (%)** | 56045 (51) | 10431 (34.2) | <0.001 |
| **SEX, Female (%)** | 54642 (49) | 15891 (52.1) | <0.001 |
| **FACILITY TYPE** | |  | <0.001 |
| Community | 10032 (11) | 1830 (8) |  |
| Comprehensive | 56263 (64) | 12847 (54) |  |
| Academic | 21813 (25) | 9011 (38) |  |
| **HOSPITAL DISTANCE >30miles (%)** | 19283 (20) | 4077 (15) | <0.001 |
| **INSURANCE STATUS (%)** | |  | <0.001 |
| None | 3411 (3) | 2241 (8) |  |
| Private | 32540 (30) | 9683 (32) |  |
| Non-private | 73723 (67) | 18145 (60) |  |
| **BELOW MEDIAN INCOME (%)** | 37898 (40) | 15021 (58) | <0.001 |
| **NEIGHBORHOOD HIGH SCHOOL EDUCATION PREVALENCE >Median(%)** | 43559 (46) | 7968 (31) | <0.001 |
| **TUMOR PRIMARY SITE, Right (%)** | 73088 (66) | 20257 (66) | 0.07 |
| **TUMOR SIZE >median (%)** | 56391 (51) | 16272 (53) | <0.001 |
| **CHEMOTHERAPY (%)** | |  | <0.001 |
| None | 75288 (68) | 19751 (65) |  |
| Neoadjuvant | 1387 (1) | 420 (1) |  |
| Adjuvant | 34388 (31) | 10356 (34) |  |
| **SURGICAL MARGINS, Positive (%)** | 6234 (6) | 1665 (6) | 0.54 |
| **CHALSON DEYO SCORE** | |  | 0.36 |
| 0 | 74453 (67) | 20337 (67) |  |
| 1 | 24388 (22) | 6768 (22) |  |
| ≥2 | 12222 (11) | 3422 (11) |  |
| **TNM STAGE (%)** | |  | <0.001 |
| 1 | 26855 (24) | 6583 (22) |  |
| 2 | 31541 (28) | 7522 (25) |  |
| 3 | 32155 (29) | 8997 (30) |  |
| 4 | 20512 (19) | 7425 (24) |  |
| **SURGICAL APPROACH (%)** | |  | <0.001 |
| MIS | 44334 (47) | 10758 (44) |  |
| MIS to open | 5734 (6) | 1616 (7) |  |
| Open | 44051 (47) | 12094 (49) |  |
| **REGIONAL NODES EXAMINED ≥12 (%)** | 87907 (80) | 22581 (75) | <0.001 |
| **READMISSION WITHIN 30-DAYS, Yes (%)** | 5203 (5) | 1487 (5) | 0.16 |
| **RURAL RESIDENTS (%)** | 21529 (20) | 4608 (15) | <0.001 |
| **NO EXCISION (%)** | 9451 (9) | 3856 (13) | <0.001 |

**Supplemental Table 4:** Clinicodemographic characteristics of Non-Hispanic White (NHW) and Black Americans diagnosed with colon cancer between 2010–2018 in the West in the NCDB.

|  | **NHW (N=46222)** | **Black**  **(N=2818)** | ***P*-value** |
| --- | --- | --- | --- |
| **AGE ≥70 (%)** | 24696 (53) | 1096 (39) | <0.001 |
| **SEX, Female (%)** | 23308 (50) | 1424 (51) | 0.93 |
| **FACILITY TYPE** |  |  | <0.001 |
| Community | 4032 (11) | 251 (11) |  |
| Comprehensive | 24651 (69) | 1228 (53) |  |
| Academic | 7096 (20) | 847 (36) |  |
| **HOSPITAL DISTANCE>30miles (%)** | 6158 (15) | 146 (6) | <0.001 |
| **INSURANCE STATUS (%)** |  |  | <0.001 |
| None | 716 (2) | 68 (2) |  |
| Private | 15263 (33) | 1057 (38) |  |
| Non-private | 29915 (65) | 1679 (60) |  |
| **BELOW MEDIAN INCOME (%)** | 8062 (20) | 837 (34) | <0.001 |
| **NEIGHBORHOOD HIGH SCHOOL EDUCATION PREVALENCE >Median(%)** | 30244 (75) | 1094 (44) | <0.001 |
| **TUMOR PRIMARY SITE, Right (%)** | 30649 (66) | 1924 (68) | 0.03 |
| **TUMOR SIZE >median (%)** | 24214 (52) | 1580 (56) | <0.001 |
| **CHEMOTHERAPY (%)** |  |  | 0.02 |
| None | 32640 (71) | 1926 (68) |  |
| Neoadjuvant | 521 (1) | 41 (2) |  |
| Adjuvant | 13061 (28) | 851 (30) |  |
| **SURGICAL MARGINS, Positive (%)** | 2712 (6) | 149 (6) | 0.57 |
| **CHALSON DEYO SCORE** |  |  | 0.16 |
| 0 | 32829 (71) | 2004 (71) |  |
| 1 | 8651 (19) | 499 (18) |  |
| ≥2 | 4742 (10) | 315 (11) |  |
| **TNM STAGE (%)** |  |  | <0.001 |
| 1 | 11691 (25) | 632 (22) |  |
| 2 | 13051 (28) | 689 (24) |  |
| 3 | 13142 (28) | 844 (30) |  |
| 4 | 8338 (18) | 653 (23) |  |
| **SURGICAL APPROACH (%)** |  |  | 0.05 |
| MIS | 20955 (54) | 1172 (51) |  |
| MIS to open | 2508 (6) | 139 (6) |  |
| Open | 15727 (40) | 977 (43) |  |
| **REGIONAL NODES EXAMINED ≥12 (%)** | 36889 (80) | 2093 (75) | <0.001 |
| **READMISSION WITHIN 30-DAYS, Yes (%)** | 1451 (3) | 66 (2) | 0.02 |
| **RURAL RESIDENTS (%)** | 4647 (10) | 16 (1) | <0.001 |
| **NO EXCISION (%)** | 4216 (9) | 388 (14) | <0.001 |

**Supplemental Table 5:** Clinicodemographic characteristics of Non-Hispanic White and Black Americans diagnosed with rectal cancer between 2010–2018 in the Northeast in the NCDB.

|  | **NHW**  **(N=19353)** | **Black**  **(N=1882)** | ***P*-value** |
| --- | --- | --- | --- |
| **AGE >70 (%)** | 8132 (42) | 670 (36) | <0.001 |
| **SEX, Female (%)** | 7814 (40) | 757 (40.) | 0.92 |
| **FACILITY TYPE** | |  | <0.001 |
| Community | 1114 (7) | 96 (6) |  |
| Comprehensive | 7050 (42) | 409 (25) |  |
| Academic | 8486 (51) | 1154 (70) |  |
| **HOSPITAL DISTANCE>30miles (%)** | 2506 (14) | 64 (4) | <0.001 |
| **INSURANCE STATUS (%)** | |  | <0.001 |
| None | 252 (1) | 63 (3) |  |
| Private | 7707 (41) | 608 (33) |  |
| Non-private | 11093 (58) | 1170 (64) |  |
| **BELOW MEDIAN INCOME (%)** | 3217 (19) | 826 (48) | <0.001 |
| **NEIGHBORHOOD HIGH SCHOOL EDUCATION PREVALENCE >Median(%)** | 12772 (74) | 527 (31) | <0.001 |
| **TUMOR SIZE >median (%)** | 10923 (56) | 1120 (60) | 0.01 |
| **CHEMOTHERAPY (%)** | |  | 0.02 |
| None | 11330 (59) | 1160 (62) |  |
| Neoadjuvant | 5638 (29) | 495 (26) |  |
| Adjuvant | 2385 (12) | 227 (12) |  |
| **RADIATION (%)** | |  | 0.03 |
| None | 12301 (64) | 1253 (67) |  |
| Neoadjuvant | 5555 (29) | 493 (26) |  |
| Adjuvant | 1497 (8) | 136 (7) |  |
| **SURGICAL MARGINS, Positive (%)** | 897 (7) | 110 (11) | <0.001 |
| **CHALSON DEYO SCORE** |  |  | 0.004 |
| 0 | 14453 (75) | 1343 (71) |  |
| 1 | 3353 (17) | 358 (19) |  |
| ≥2 | 1547 (8) | 181 (10) |  |
| **TNM STAGE (%)** | |  | <0.001 |
| 1 | 6168 (32) | 481 (26) |  |
| 2 | 4448 (23) | 419 (22) |  |
| 3 | 5040 (26) | 553 (29) |  |
| 4 | 3697 (19) | 429 (23) |  |
| **SURGICAL APPROACH (%)** | |  | 0.001 |
| MIS | 6081 (53) | 463 (47) |  |
| MIS to open | 735 (6) | 63 (6) |  |
| Open | 4584 (40) | 452 (46) |  |
| **REGIONAL NODES EXAMINED ≥12 (%)** | 7945 (42) | 640 (35) | <0.001 |
| **READMISSION WITHIN 30-DAYS, Yes (%)** | 901 (5) | 107 (6) | <0.05 |
| **RURAL RESIDENTS (%)** | 1877 (10) | 14 (1) | <0.001 |
| **NO EXCISION (%)** | 6147 (33) | 776 (42) | <0.001 |

**Supplemental Table 6:** Clinicodemographic characteristics of Non-Hispanic White and Black Americans diagnosed with rectal cancer between 2010–2018 in the Midwest in the NCDB.

|  | **NHW**  **(N=26424)** | **Black (N=2130)** | ***P*-value** |
| --- | --- | --- | --- |
| **AGE >70 (%)** | 10400 (39) | 698 (33) | <0.001 |
| **SEX, Female (%)** | 10158 (38) | 886 (42) | <0.01 |
| **FACILITY TYPE** |  |  | <0.001 |
| Community | 2617 (13) | 127 (8) |  |
| Comprehensive | 9024 (46) | 456 (29) |  |
| Academic | 8143 (41) | 1007 (63) |  |
| **HOSPITAL DISTANCE>30miles (%)** | 6370 (27) | 99 (5) | <0.001 |
| **INSURANCE STATUS (%)** |  |  | <0.001 |
| None | 639 (2) | 86 (4) |  |
| Private | 10018 (38) | 656 (32) |  |
| Non-private | 15450 (59) | 1336 (64) |  |
| **BELOW MEDIAN INCOME (%)** | 5896 (26) | 1081 (57) | <0.001 |
| **NEIGHBORHOOD HIGH SCHOOL EDUCATION PREVALENCE >Median(%)** | 16618 (72) | 666 (35) | <0.001 |
| **TUMOR SIZE >median (%)** | 14697 (56) | 1263 (59) | 0.001 |
| **CHEMOTHERAPY (%)** | |  | <0.001 |
| None | 14794 (56) | 1290 (61) |  |
| Neoadjuvant | 8120 (31) | 598 (28) |  |
| Adjuvant | 3510 (13) | 242 (11) |  |
| **RADIATION (%)** | |  | 0.001 |
| None | 15967 (60) | 1369 (64) |  |
| Neoadjuvant | 8245 (31) | 616 (29) |  |
| Adjuvant | 2212 (8) | 145 (7) |  |
| **SURGICAL MARGINS, Positive (%)** | 1319 (7) | 113 (9) | 0.03 |
| **CHALSON DEYO SCORE** |  |  | 0.36 |
| 0 | 19079 (72) | 1516 (71) |  |
| 1 | 4929 (19) | 400 (19) |  |
| ≥2 | 2416 (9) | 214 (10) |  |
| **TNM STAGE (%)** | |  | <0.001 |
| 1 | 8517 (32) | 573 (27) |  |
| 2 | 5998 (23) | 471 (22) |  |
| 3 | 6908 (26) | 575 (27) |  |
| 4 | 5001 (19) | 511 (24) |  |
| **SURGICAL APPROACH (%)** | |  | <0.001 |
| MIS | 8274 (50) | 535 (44) |  |
| MIS to open | 864 (5) | 77 (6) |  |
| Open | 7528 (45) | 607 (50) |  |
| **REGIONAL NODES EXAMINED ≥12 (%)** | 12184 (47) | 822 (40) | <0.001 |
| **READMISSION WITHIN 30-DAYS, Yes (%)** | 1293 (5) | 101 (5) | 0.76 |
| **RURAL RESIDENTS (%)** | 7222 (28) | 64 (3) | <0.001 |
| **NO EXCISION (%)** | 7122 (28) | 772 (38) | <0.001 |

**Supplemental Table 7:** Clinicodemographic characteristics of Non-Hispanic White and Black Americans diagnosed with rectal cancer between 2010–2018 in the South in the NCDB.

|  | **NHW**  **(N=32692)** | **Black**  **(N=6381)** | ***P*-value** |
| --- | --- | --- | --- |
| **AGE >70 (%)** | 11802 (36) | 1862 (29) | <0.001 |
| **SEX, Female (%)** | 12560 (38) | 2562 (40) | 0.01 |
| **FACILITY TYPE** |  |  | <0.001 |
| Community | 2404 (9) | 328 (7) |  |
| Comprehensive | 14659 (56) | 2442 (48) |  |
| Academic | 9264 (35) | 2309 (46) |  |
| **HOSPITAL DISTANCE>30miles (%)** | 8065 (28) | 1126 (20) | <0.001 |
| **INSURANCE STATUS (%)** |  |  | <0.001 |
| None | 1482 (5) | 593 (10) |  |
| Private | 12247 (38) | 1995 (32) |  |
| Non-private | 18371 (57) | 3670 (59) |  |
| **BELOW MEDIAN INCOME (%)** | 11803 (43) | 3210 (59) | <0.001 |
| **NEIGHBORHOOD HIGH SCHOOL EDUCATION PREVALENCE >Median(%)** | 11918 (43) | 1538 (28) | <0.001 |
| **TUMOR SIZE >median (%)** | 18381 (56) | 3770 (59) | <0.001 |
| **CHEMOTHERAPY (%)** | |  | <0.001 |
| None | 17799 (54) | 3789 (59) |  |
| Neoadjuvant | 10335 (32) | 1786 (28) |  |
| Adjuvant | 4558 (14) | 806 (13) |  |
| **RADIATION (%)** | |  | <0.001 |
| None | 19618 (60) | 4056 (64) |  |
| Neoadjuvant | 10110 (31) | 1761 (28) |  |
| Adjuvant | 2964 (9) | 564 (9) |  |
| **SURGICAL MARGINS, Positive (%)** | 1808 (8) | 395 (10) | <0.001 |
| **CHALSON DEYO SCORE** |  |  | <0.001 |
| 0 | 24339 (74) | 4610 (72) |  |
| 1 | 5911 (18) | 1205 (19) |  |
| ≥2 | 2442 (8) | 566 (9) |  |
| **TNM STAGE (%)** | |  | <0.001 |
| 1 | 10497 (32) | 1710 (27) |  |
| 2 | 7644 (23) | 1452 (23) |  |
| 3 | 8515 (26) | 1719 (27) |  |
| 4 | 6036 (19) | 1500 (24) |  |
| **SURGICAL APPROACH (%)** | |  | <0.01 |
| MIS | 9418 (45) | 1480 (42) |  |
| MIS to open | 1016 (5) | 189 (5) |  |
| Open | 10310 (50) | 1840 (52) |  |
| **REGIONAL NODES EXAMINED ≥12 (%)** | 14324 (45) | 2366 (38) | <0.001 |
| **READMISSION WITHIN 30-DAYS, Yes (%)** | 1325 (4) | 279 (4) | 0.25 |
| **RURAL RESIDENTS (%)** | 7206 (23) | 1071 (17) | <0.001 |
| **NO EXCISION (%)** | 8909 (28) | 2331 (38) | <0.001 |

**Supplemental Table 8:** Clinicodemographic characteristics of Non-Hispanic White and Black Americans diagnosed with rectal cancer between 2010–2018 in the West in the NCDB.

|  | **NHW**  **(N=13957)** | **Black**  **(N=603)** | ***P*-value** |
| --- | --- | --- | --- |
| **AGE >70 (%)** | 5162 (37) | 169 (28) | <0.001 |
| **SEX, Female (%)** | 5552 (40) | 249 (41) | 0.48 |
| **FACILITY TYPE** |  |  | <0.001 |
| Community | 1025 (10) | 60 (12) |  |
| Comprehensive | 6648 (62) | 197 (41) |  |
| Academic | 3024 (28) | 226 (47) |  |
| **HOSPITAL DISTANCE>30miles (%)** | 2818 (22) | 44 (8) | <0.001 |
| **INSURANCE STATUS (%)** |  |  | <0.01 |
| None | 292 (2) | 22 (4) |  |
| Private | 5870 (42) | 227 (38) |  |
| Non-private | 7685 (56) | 352 (59) |  |
| **BELOW MEDIAN INCOME (%)** | 2703 (22) | 183 (35) | <0.001 |
| **NEIGHBORHOOD HIGH SCHOOL EDUCATION PREVALENCE >Median(%)** | 8801 (73) | 216 (41) | <0.001 |
| **TUMOR SIZE >median (%)** | 7789 (56) | 351 (58) | 0.26 |
| **CHEMOTHERAPY (%)** | |  | 0.12 |
| None | 8215 (59) | 379 (63) |  |
| Neoadjuvant | 3952 (28) | 159 (26) |  |
| Adjuvant | 1790 (13) | 65 (11) |  |
| **RADIATION (%)** | |  | 0.57 |
| None | 8895 (64) | 397 (66) |  |
| Neoadjuvant | 3886 (28) | 158 (26) |  |
| Adjuvant | 1176 (8) | 48 (8) |  |
| **SURGICAL MARGINS, Positive (%)** | 784 (8) | 35 (10) | 0.33 |
| **CHALSON DEYO SCORE** |  |  | 0.82 |
| 0 | 10836 (78) | 473 (78) |  |
| 1 | 2194 (16) | 89 (15) |  |
| ≥2 | 927 (7) | 41 (7) |  |
| **TNM STAGE (%)** | |  | <0.01 |
| 1 | 4413 (32) | 177 (29) |  |
| 2 | 3136 (23) | 111 (18) |  |
| 3 | 3734 (27) | 167 (28) |  |
| 4 | 2674 (19) | 148 (25) |  |
| **SURGICAL APPROACH (%)** | |  | 0.22 |
| MIS | 4713 (55) | 182 (55) |  |
| MIS to open | 476 (6) | 26 (8) |  |
| Open | 3322 (39) | 124 (37) |  |
| **REGIONAL NODES EXAMINED ≥12 (%)** | 6005 (44) | 227 (38) | 0.01 |
| **READMISSION WITHIN 30-DAYS, Yes (%)** | 402 (3) | 17 (3) | 1 |
| **RURAL RESIDENTS (%)** | 1780 (13) | 6 (1) | <0.001 |
| **NO EXCISION (%)** | 4142 (30) | 225 (37) | <0.001 |

**Supplemental Table 9:** Multivariable model of factors associated with colon cancer mortality among NHW patients treated across the four US regions.

|  | **Variables** | **Hazard Ratio (95% CI)** | ***P*-value** |
| --- | --- | --- | --- |
| **AGE** | <70 | *Ref* |  |
|  | ≥70 | 1.74 (1.71–1.77) | <0.001 |
| **SEX** | Male | *Ref* |  |
|  | Female | 0.93 (0.92–0.94) | <0.001 |
| **METRO** | Urban | *Ref* |  |
|  | Rural | 1.00 (0.98–1.03) | 0.75 |
| **FACILITY TYPE** | Community | *Ref* |  |
|  | Comprehensive | 1.00 (0.98–1.02) | 0.99 |
|  | Academic | 0.89 (0.87–0.91) | <0.001 |
| **CHALSON DEYO SCORE** | 0 | *Ref* |  |
|  | 1 | 1.21 (1.19–1.23) | <0.001 |
|  | ≥2 | 1.61 (1.58–1.64) | <0.001 |
| **INSURANCE** | None | *Ref* |  |
|  | Private | 0.71 (0.67–0.74) | <0.001 |
|  | Government | 0.94 (0.89–0.99) | 0.01 |
| **HOSPITAL DISTANCE** | <30 miles | *Ref* |  |
|  | ≥30 miles | 0.98 (0.96–1.01) | 0.17 |
| **INCOME** | ≥ Median | *Ref* |  |
|  | < Median | 1.09 (1.07–1.11) | <0.001 |
| **TUMOR SIZE** | <Median | *Ref* |  |
|  | ≥Median | 1.06 (1.04–1.07) | <0.001 |
| **NUMBER OF NODES EXAMINED** | <12 | *Ref* |  |
|  | ≥12 | 0.68 (0.66–0.69) | <0.001 |
| **TNM STAGE** | 1 | *Ref* |  |
|  | 2 | 1.53 (1.49–1.57) | <0.001 |
|  | 3 | 3.26 (3.18–3.34) | <0.001 |
|  | 4 | 9.99 (9.71–10.3) | <0.001 |
| **TUMOR EXCISION** | Yes | *Ref* |  |
|  | No | 1.14 (1.11–1.18) | <0.001 |
| **CHEMOTHERAPY** | None | *Ref* |  |
|  | Neoadjuvant | 0.59 (0.55–0.63) | <0.001 |
|  | Adjuvant | 0.51 (0.50–0.52) | <0.001 |
| **READMISSION** | No |  |  |
|  | Yes | 1.46 (1.41–1.50) | <0.001 |
| **GEOGRAPHIC REGION** | Northeast | *Ref* |  |
|  | Midwest | 1.09 (1.07–1.11) | <0.001 |
|  | South | 1.03 (1.01–1.05) | <0.01 |
|  | West | 0.99 (0.97–1.02) | 0.69 |

**Supplemental Table 10**: Multivariable model of factors associated with rectal cancer mortality among NHW patients treated across the four US regions.

|  | **Variables** | **Hazard Ratio (95% CI)** | ***P*-value** |
| --- | --- | --- | --- |
| **AGE** | <70 | *Ref* |  |
|  | ≥70 | 1.78 (1.73–1.84) | <0.001 |
| **SEX** | Male | *Ref* |  |
|  | Female | 0.94 (0.92–0.97) | <0.001 |
| **METRO** | Urban | *Ref* |  |
|  | Rural | 1.03 (0.99–1.07) | 0.17 |
| **FACILITY TYPE** | Community | *Ref* |  |
|  | Comprehensive | 0.99 (0.95–1.04) | 0.76 |
|  | Academic | 0.86 (0.82–0.90) | <0.001 |
| **CHALSON DEYO SCORE** | 0 | *Ref* |  |
|  | 1 | 1.24 (1.20–1.28) | <0.001 |
|  | ≥2 | 1.77 (1.69–1.85) | <0.001 |
| **INSURANCE** | None | *Ref* |  |
|  | Private | 0.65 (0.60–0.70) | <0.001 |
|  | Government | 0.93 (0.87–1.00) | 0.05 |
| **HOSPITAL DISTANCE** | <30 miles | *Ref* |  |
|  | ≥30 miles | 0.96 (0.92–0.99) | 0.02 |
| **INCOME** | ≥ Median | *Ref* |  |
|  | < Median | 1.07 (1.03–1.10) | <0.001 |
| **TUMOR SIZE** | <Median | *Ref* |  |
|  | ≥Median | 1.08 (1.05–1.11) | <0.001 |
| **NUMBER OF NODES EXAMINED** | <12 | *Ref* |  |
|  | ≥12 | 0.83 (0.80–0.86) | <0.01 |
| **TNM STAGE** | 1 | *Ref* |  |
|  | 2 | 1.65 (1.58–1.73) | <0.001 |
|  | 3 | 1.99 (1.91–2.01) | <0.001 |
|  | 4 | 5.04 (4.80–5.29) | <0.001 |
| **TUMOR EXCISION** | Yes | *Ref* |  |
|  | No | 1.79 (1.71–1.89) | <0.001 |
| **CHEMOTHERAPY** | None | *Ref* |  |
|  | Neoadjuvant | 0.82 (0.76–0.89) | <0.001 |
|  | Adjuvant | 0.75 (0.71–0.80) | <0.01 |
| **RADIATION** | None | *Ref* |  |
|  | Neoadjuvant | 0.91 (0.85–0.98) | 0.02 |
|  | Adjuvant | 0.95 (0.89–1.02) | 0.16 |
| **READMISSION** | No |  |  |
|  | Yes | 1.26 (1.17–1.35) | <0.001 |
| **GEOGRAPHIC REGION** | Northeast | *Ref* |  |
|  | Midwest | 1.15 (1.11–1.20) | <0.001 |
|  | South | 1.11 (1.07–1.16) | <0.001 |
|  | West | 1.07 (1.02–1.12) | <0.01 |
